# Editorial

**Published:** 1872-04-01

**Authors:** 


### MEDICAL EXAMINER.

*A Semi-Monthly Journal of Medical Sciences.*

EDITED BY

N. S. DAVIS, M. D., AND F. H. DAVIS, M. D.

Chicago, April 1st, 1872.

### EDITORIAL.

ASSOCIATION OF AMERICAN MEDICAL EDITORS.—This organization will hold its third Anniversary meeting in Philadelphia, Monday, May 6th, 1872. The members will assemble for the transaction of business at 11 o'clock A. M., and the President, Dr. B. F. Dawson, of New York, will deliver the annual address at 8 o'clock in the evening. The meetings will probably be held in Horticultural Hall, on Broad street.

AMERICAN MEDICAL ASSOCIATION.—Delegates from the Northwest will find the Pittsburg, Fort Wayne and Chicago, and the Pennsylvania Central railroads the most direct route to Philadelphia, on which excursion tickets can be purchased on presenting at the ticket offices the certificates obtained from the Permanent Secretary, Dr. W. B. Atkinson, 1400 Pine street, Philadelphia. The excursion tickets can be obtained from the 1st to the 7th of May, and will be good until the 20th of the same month.

ILLINOIS STATE MEDICAL SOCIETY.—The next annual meeting of this society will be held in Rock Island, commencing on the 3d Tuesday (21st) in May. We hope it will be borne in mind by the profession throughout the State, and that there will be a full attendance. We are sure our friends in Rock Island will give the members a cordial reception.

THE TONER LECTURES.—We are gratified to see by a Washington paper that our friend Dr. J. M. Toner, of Washington, D. C., has placed in the hands of Trustees, property to the amount of $3,000, the interest on which is to be applied to the annual payment of premiums for two essays or memoirs, to be read in that city and published in such way as the Trustees shall deem proper. The object is to advance medical science and a knowledge of the laws of nature by encouraging thorough original investigation. No essay or lecture is to be accepted by the Trustees unless it embodies some actual addition to the knowledge previously existing concerning the same subject; and this is to be determined by a critical examination to be made by a competent committee. The example is worthy of imitation.

CHICAGO MEDICAL COLLEGE.—The spring and summer course of instruction in this institution is well attended; and the clinical advantages are not excelled in any other city.

MEDICAL REGISTER AND DIRECTORY OF THE UNITED STATES.—Dr. J. M. Toner, of Washington, D. C., is diligently preparing a Register of the medical practitioners of the United States, which is to be published by S. W. Butler, M. D., of Philadelphia. The work is a very important one,. and to insure accuracy in the list of names, many thousand circulars have been sent to physicians all over the country. Those to whom the circulars have been sent should promptly return them with the information asked, as it will greatly facilitate the appearance of the work. The task of the author and publisher is an arduous one, and the work one to be desired by every member of the profession.

SMALL-POX AND MEASLES.—In another part of the present number of the EXAMINER Prof. Bevan reports a case presenting a curious mixture of the symptoms of scarlatina, measles and small-pox.

Two similar cases have recently come under our own observation. Both were adults of foreign birth.

Both were attacked with all the symptoms of measles in a severe form. The coryza, bronchial cough, soreness of throat, and fever were strongly marked; and about the fourth day a very copious exanthematous rash appeared over the whole surface, the fever and catarrhal symptoms continuing unabated. In one of them the fever from the beginning presented a strongly typhoid character, the pulse being frequent and weak; the mind wandering; and the tongue and mouth dry.

The eruption was so thick as to make the whole surface deep red; and there appeared co-incidently on the body and lower extremities numerous small petechial or hemorrhagic spots. In about three days from the appearance of the eruption, the face and nearly all the surface became swollen as in small-pox, and emitted the same odor, while the hemorrhagic spots became larger, more numerous, the pulse more rapid and feeble, the mind constantly delirious; and the following day there came hemorrhage from the bowels, from the mucous membrane of the mouth and nostrils, with rapid collapse and death. The other case, which occurred at the same time and in the same room, presented precisely the same character of symptoms, only less severe, and only slight hemorrhagic spots. The catarrhal symptoms of measles, the exanthematous character of the eruptions were perfect; yet at the ordinary time the swelling of the face and the odor perfectly resembled small-pox; and there gradually developed scattered pimples of larger size, flattened, but neither umbilicated nor vesicular. They appeared like large flattened papules interspersed with the exanthematous rash. At this stage the case was removed to the small-pox hospital, and we did not see its subsequent progress.

